# The Pathophysiology of Acute Coronary Syndrome

**DOI:** 10.31083/j.rcm2407213

**Published:** 2023-07-21

**Authors:** Salvatore De Rosa, Daniele Torella, Isabella Leo

**Affiliations:** ^1^Department of Medical and Surgical Sciences, Magna Graecia University, 88100 Catanzaro, Italy; ^2^Department of Experimental and Clinical Medicine, Magna Graecia University, 88100 Catanzaro, Italy; ^3^CMR Unit, Royal Brompton and Harefield Hospital, UB9 6JH London, UK

Despite the remarkable advances in the prevention, diagnosis and treatment of cardiovascular disease, coronary artery disease (CAD) still remains a leading cause of mortality worldwide [1]. Acute coronary syndromes (ACS) account for most of its morbidity and mortality burden [2], stimulating more in-depth research on the underlying mechanisms. This special issue collects original studies shedding new light on pathophysiology of ACS, along with systematic state-of-art reviews on key topics, highlighting knowledge gaps research should point at. Novel interest is emerging on classic concepts, including total plaque burden, high-risk plaque features and inflammation, as described in a comprehensive review highlighting key evolving concepts in the pathophysiology of ACS [3, 4, 5]. Takotsubo syndrome (TTS) usually presents with signs and symptoms in overlap with ACS [6]. Frequently triggered by an emotional stressor, cases of TTS have recently been reported after pacemaker implantation (PMI). Strangio *et al*. [7], provide for the first time a comprehensive report of the clinical picture of TTS after PMI, proposing possible strategies for early detection. This diagnostic conundrum is particularly relevant, as bradyarrhythmia can go along with ACS events. In this regard, temporary PMI is reported in approximately 1% of patients, assuming prognostic relevance, as shown in this issue by a 10-years historical multicenter cohort study involving 35,394 patients [8].

Among the clinical hurdles with ACS, early diagnosis and differentiation from acute heart failure of different etiologies is particularly relevant but can be sometimes challenging. The clinical presentation of acute aortic dissection has some overlap, especially when associated to hypoperfusion of multiple tissues and organs. In this regard, a systematic revision of all cases published to date included in this issue, provides a useful picture of the rare, yet dreadful condition of acute type A aortic dissection with mesenteric malperfusion, associated to a high in-hospital mortality [9].

Myocardial infarction with non-obstructive coronary arteries (MINOCA) is known to be very common, with affected patients more frequently being young, female, presenting a lower-than-expected burden of classical cardiovascular (CV) risk factors. Other than a homogeneous nosological entity, MINOCA encompasses several atherosclerotic and non-atherosclerotic causes. This heterogeneity often represents a hassle to pursue the etiological diagnosis and requires multiple invasive and non-invasive tests. This special issue features a comprehensive overview on the topic, aiming at individuating clinical signs and symptoms of prognostic relevance [10]. The authors also provided a comprehensive review of the intracoronary imaging and coronary physiological techniques useful in this setting, that are currently a key research focus in this area [11, 12]. These novel tools complement standard coronary angiography, providing additional information about the culprit lesion and identifying high risk plaque features. A thorough knowledge of their strengths and limitations is key to better understand the most appropriate diagnostic approach [13]. Advanced imaging and angiographic modalities represent only one possible approach to identify high-risk lesions. There is in fact growing interest about the potential role of novel circulating biomarkers of instability [14]. In this regard, plasma levels of trimethylamine N-Oxide (TMAO) are independently associated with plaque rupture and can be helpful to distinguish it from plaque erosion [4, 15]. The latter has been instead associated with elevated levels of Neutrophil extracellular traps (NETs) and Interleukin-8 (IL-8).

The immune system and the pro-inflammatory environment play an unquestionable role in patients with ACS, with possible therapeutic implications. One clear example is the evidence that Canakinumab has proven to reduce CV events in patients with prior myocardial infarction (MI), inhibiting the IL-1 mediated inflammatory response [16]. In this issue, Liu *et al*. [17] report that lower levels of circulating soluble interleukin-1 receptor type 2 (sIL-1R2) were associated with an increased risk of worsened LVEF and adverse clinical outcome at 12 months. In addition, regulatory T-cells have a lower count in ST Elevation Myocardial Infarction (STEMI) patients undergoing percutaneous coronary intervention (PCI) and accurately predict the presence of intramyocardial hemorrhage [18]. An emerging yet powerful tool to detect inflammation nowadays is cardiac imaging. The pericoronary fat attenuation index (pFAI) is in fact a computed tomography biomarker of coronary inflammation, correlated with increased risk of cardiac mortality. The authors found higher pFAI values in diabetic patients with poorer glycemic control [19]. This could represent an additional tool, to identify patients at higher risk of future CV events. Vascular inflammation is a key element in the response of the vessel wall to vascular interventions, more so when a relevant traumatic stimulus is present. Using an approach at the forefront of biomedical technologies, the authors assessed the long-term results of a novel covered stent, useful to treat the dreadful complication of coronary perforation equipped with a biodegradable membrane. This might help to counteract the risks of restenosis and thrombosis, which are usually frequently observed with covered stents [20].

The advent of novel anti-thrombotic drugs has drastically changed the landscape of ACS outcomes. Treatment with more recent P2Y12 inhibitors has in fact proven to be superior to clopidogrel in preventing ischemic CV events. However, the undeniable benefit coming from the use of these potent drugs has been questioned by the increased risk of bleeding events, with potential risk of discontinuation [21]. A sub-analysis of the IDEAL-LDL trial published in this special issue tried to address this concern, demonstrating that potency of P2Y12 inhibitors does not affect adherence to therapy [22]. Randomized trials demonstrated the superiority of complete revascularization over culprit-only revascularization in patients with STEMI, even though this debate is still open. In this context, a metanalysis including 2256 patients showed no significant difference in clinical outcomes among the two strategies, suggesting a tailored approach when dealing with ACS patients with multivessel disease [23].

When dealing with ACS, several are therefore the aspects that merit further investigation, with novel concepts emerging in the last decades. A terse identification of currently unmet clinical needs and a thorough comprehension of the complex pathophysiological mechanisms subtended to ACS are the necessary starting point to fuel the development of new therapeutic and preventive strategies. The articles collected in this special issue sound a bell to some underestimated issues, representing a first step in the appropriate direction, hopefully meeting the need of dedicated literature on this never outdated topic.

## References

[1] Roth GA, Johnson C, Abajobir A, Abd-Allah F, Abera SF, Abyu G (2017). Global, Regional, and National Burden of Cardiovascular Diseases for 10 Causes, 1990 to 2015. *Journal of the American College of Cardiology*.

[2] Puymirat E, Simon T, Cayla G, Cottin Y, Elbaz M, Coste P (2017). Acute Myocardial Infarction: Changes in Patient Characteristics, Management, and 6-Month Outcomes Over a Period of 20 Years in the FAST-MI Program (French Registry of Acute ST-Elevation or Non-ST-Elevation Myocardial Infarction) 1995 to 2015. *Circulation*.

[3] Stone PH, Libby P, Boden WE (2023). Fundamental Pathobiology of Coronary Atherosclerosis and Clinical Implications for Chronic Ischemic Heart Disease Management—The Plaque Hypothesis: A Narrative Review. *JAMA Cardiology*.

[4] Yuan D, Chu J, Qian J, Lin H, Zhu G, Chen F (2023). New Concepts on the Pathophysiology of Acute Coronary Syndrome. *Reviews in Cardiovascular Medicine*.

[5] Kazem N, Hofer F, Koller L, Hammer A, Hofbauer TM, Hengstenberg C (2021). The age-specific prognostic impact of the platelet-to-lymphocyte ratio on long-term outcome after acute coronary syndrome. *European Heart Journal Open*.

[6] Matta A, Delmas C, Campelo-Parada F, Lhermusier T, Bouisset F, Elbaz M (2022). Takotsubo cardiomyopathy. *Reviews in Cardiovascular Medicine*.

[7] Strangio A, Leo I, Sabatino J, Romano LR, Critelli C, Canino G (2022). Takotsubo Syndrome after Pacemaker Implantation: A Systematic Review. *Reviews in Cardiovascular Medicine*.

[8] Wang P, Wang S, Liu Z, Song L, Xu B, Dou K (2023). Prognostic Value of Temporary Pacemaker Insertion in Patients with Acute Myocardial Infarction in the Era of Percutaneous Coronary Revascularization. *Reviews in Cardiovascular Medicine*.

[9] Wang C, Wu H, Xi Z, Liu Q, Sun L, Zhang L (2023). Systematic Review of the Management of Acute Type A Aortic Dissection with Mesenteric Malperfusion. *Reviews in Cardiovascular Medicine*.

[10] Scagliola R, Senes J, Balbi M (2022). Myocardial Infarction with Non-Obstructive Coronary Arteries: A Puzzle in Search of a Solution. *Reviews in Cardiovascular Medicine*.

[11] Usui E, Matsumura M, Smilowitz NR, Mintz GS, Saw J, Kwong RY (2022). Coronary morphological features in women with non-ST-segment elevation MINOCA and MI-CAD as assessed by optical coherence tomography. *European Heart Journal Open*.

[12] van Veelen A, van der Sangen NMR, Henriques JPS, Claessen BEPM (2022). Identification and treatment of the vulnerable coronary plaque. *Reviews in Cardiovascular Medicine*.

[13] Sun Q, Liu M, Zeng M, Jia H (2023). Intracoronary Diagnostics in Patients with Acute Coronary3Syndrome. *Reviews in Cardiovascular Medicine*.

[14] De Rosa S, Indolfi C, Igaz P (2015). Circulating microRNAs as Biomarkers in Cardiovascular Diseases. *Circulating microRNAs in Disease Diagnostics and their Potential Biological Relevance. Experientia Supplementum*.

[15] Zhang F, Li B, Su H, Guo Z, Zhu H, Wang A (2023). Progress in the metabolomics of acute coronary syndrome. *Reviews in Cardiovascular Medicine*.

[16] Ridker PM, Everett BM, Thuren T, MacFadyen JG, Chang WH, Ballantyne C (2017). Antiinflammatory Therapy with Canakinumab for Atherosclerotic Disease. *The New England Journal of Medicine*.

[17] Liu SF, Liu S, Yu QT, Gao TG, Zhang Y, Cai JY (2022). Association of Soluble IL-1 Receptor Type 2 with Recovery of Left Ventricular Function and Clinical Outcomes in Acute Myocardial Infarction. *Reviews in Cardiovascular Medicine*.

[18] Zhang Y, Gao H, Liu L, Li S, Hua B, Lan D (2023). Regulatory T cell as Predictor of Intramyocardial Hemorrhage in STEMI Patients after primary PCI. *Reviews in Cardiovascular Medicine*.

[19] Jiang J, Yin Y, Li Y, Xu B, Zou Z, Ding S (2023). Impact of Glycemic Control on Coronary Inflammation evaluated by Computed Tomography Pericoronary Fat Attenuation Index in Patients with Acute Coronary Syndrome. *Reviews in Cardiovascular Medicine*.

[20] Cai W, Chen E, Zheng H, Hu D, Wu L, Zeng X (2023). Mid-term outcomes of novel covered stent with biodegradable membrane in porcine coronary artery perforation. *Reviews in Cardiovascular Medicine*.

[21] Sabouret P, Spadafora L, Fischman D, Ullah W, Zeitouni M, Gulati M (2022). De-escalation of antiplatelet therapy in patients with coronary artery disease: Time to change our strategy. *European Journal of Internal Medicine*.

[22] Kourti O, Konstantas O, Farmakis IΤ, Zafeiropoulos S, Psarakis G, Vrana E (2022). Potent P2Y12 inhibitors versus clopidogrel to predict adherence to antiplatelet therapy after an acute coronary syndrome: insights from IDEAL-LDL. *Reviews in Cardiovascular Medicine*.

[23] Panuccio G, Salerno N, De Rosa S, Torella D (2023). Timing of Complete Revascularization in Patients with STEMI and Multivessel Disease: A Systematic Review and Meta-Analysis. *Reviews in Cardiovascular Medicine*.

